# Epidermolysis bullosa acquisita^⋆^

**DOI:** 10.1016/j.abd.2021.09.010

**Published:** 2022-06-11

**Authors:** Denise Miyamoto, Juliana Olivieri Gordilho, Claudia Giuli Santi, Adriana Maria Porro

**Affiliations:** a Department of Dermatology, Hospital das Clínicas, Faculty of Medicine, Universidade de São Paulo, São Paulo, SP, Brazil; b Department of Dermatology, Escola Paulista de Medicina, Universidade Federal de São Paulo, São Paulo, SP, Brazil

**Keywords:** Autoimmunity, Epidermolysis bullosa acquisita, Vesiculobullous skin diseases

## Abstract

Epidermolysis bullosa acquisita is a rare autoimmune disease, characterized by the synthesis of anti-collagen VII autoantibodies, the main component of hemidesmosome anchoring fibrils. The antigen-antibody binding elicits a complex inflammatory response, which culminates in the loss of dermo-epidermal adhesion of the skin and/or mucous membranes. Skin fragility with bullae, erosions, and milia in areas of trauma characterizes the mechanobullous form of the disease. In the inflammatory form of epidermolysis bullosa acquisita, urticarial inflammatory plaques with tense bullae, similar to bullous pemphigoid, or mucosal lesions can determine permanent scars and loss of functionality in the ocular, oral, esophageal, and urogenital regions. Due to the similarity of the clinical findings of epidermolysis bullosa acquisita with other diseases of the pemphigoid group and with porphyria cutanea tarda, the diagnosis is currently confirmed mainly based on the clinical correlation with histopathological findings (pauci-inflammatory subepidermal cleavage or with a neutrophilic infiltrate) and the demonstration of the presence of anti-collagen VII IgG *in situ* by direct immunofluorescence, or circulating anti-collagen VII IgG through indirect immunofluorescence and/or ELISA. There is no specific therapy for epidermolysis bullosa acquisita and the response to treatment is variable, usually with complete remission in children and a worse prognosis in adults with mucosal involvement. Systemic corticosteroids and immunomodulators (colchicine and dapsone) are alternatives for the treatment of mild forms of the disease, while severe forms require the use of corticosteroid therapy associated with immunosuppressants, intravenous immunoglobulin, and rituximab.

## Introduction and history

Epidermolysis bullosa acquisita (EBA) is a rare autoimmune dermatosis triggered by autoantibodies against collagen VII (COLVII), the main component of the anchoring fibrils of the stratified squamous epithelium. The resulting loss of dermo-epidermal adhesion can manifest from mild skin fragility to severe mucosal stenosis.

The first report of EBA occurred in 1895 when Elliot (1895, *apud* ROENIGK, 1971, p.1) described two adults with acquired skin fragility.^1^ Additional cases of EBA were published in subsequent years. However, as the diagnosis was mainly based on mucocutaneous characteristics, it was not possible to rule out other differential diagnoses, such as porphyria cutanea tarda and bullous pemphigoid.^1^ In 1965, Pass et al. performed histochemical studies and suggested that EBA pathogenesis was related to collagen alterations.^2^

The initial diagnostic criteria were only established in 1971 by Roenigk et al.^1^ The autoimmune nature of EBA was demonstrated by the presence of IgG deposits in the basement membrane zone (BMZ) using direct immunofluorescence evaluation.^3^ The exact location of immune complex deposits in the lamina densa was clarified by Yaoita et al.^4^ and Nieboer et al.^5^ using immuno-electron microscopy, and a 290 kDa protein – collagen VII – was identified by Woodley et al. in 1984 as the target antigen in EBA.^6^

More recent reports of patients with inflammatory lesions of the bullous pemphigoid type,^7^ or with predominant mucosal involvement similar to mucous membrane pemphigoid, reinforce the need for laboratory tests to demonstrate the presence of anti-COLVII autoantibodies^8^ for diagnostic confirmation of EBA and differentiation from the pemphigoid group.

## Epidemiology

The annual incidence of EBA is estimated to range from 0.08 to 0.5 cases per million individuals,^9^, ^10^ corresponding to approximately 5% of cases of patients with antibodies against the basement membrane zone.^11^

EBA has no sex predilection and its onset usually occurs between the fourth and fifth decades of life.^12^ However, individuals of any age can be affected. A recent meta-analysis revealed that, among patients diagnosed with EBA, 4.6% were younger than 17 years.^13^ Childhood EBA occurs between two weeks to 17 years of age.^14^ The inflammatory clinical form is the most frequent one and is usually accompanied by mucosal lesions.^14^ A neonatal form resulting from the placental transfer of maternal autoantibodies has been described in EBA.^15^

## Etiopathogenesis

EBA is an autoimmune disease that belongs to the group of subepidermal bullous dermatoses. Its main antigenic target is COLVII, located in the sublamina densa of the BMZ. COLVII, the main component of the anchoring fibrils, is a 290 kDa protein that consists of a central collagenous domain flanked by two non-collagenous domains, NC1 and NC2.^8^ In patients with EBA, most autoantibodies target epitopes located in the NC1 domain, although reactivity against the collagenous or NC2 domains can be detected in a minority of cases.^16^ In general, these autoantibodies are of the IgG type. However, IgA, IgE, and IgM have been detected in some patients.^7^

Experimental studies suggest that genetic susceptibility to the disease is especially associated with HLA-DR2. More recently, evidence of the involvement of genes that do not belong to the major histocompatibility complex (MHC) has been described in experimental models of EBA.^7^ Additional studies have demonstrated the protective role of the skin microbiota diversity in the clinical manifestations of EBA.^17^, ^18^

In animal-induced inflammatory EBA, T-cell-deficient mice do not develop specific autoantibodies against COLVII, demonstrating the participation of T-lymphocytes in the disease pathogenesis. Regulatory T-cells also play a protective role in EBA development.^7^ The sensitization of CD4 + T lymphocytes requires the presence of antigen-presenting cells (APCs), with their flow in peripheral lymph nodes mediated by the granulocyte-macrophage colony-stimulating factor (GM-CSF) and by neutrophils.^19^ In addition to APCs, dendritic cells, macrophages, and B lymphocytes are required for clonal expansion and plasma cell differentiation, with subsequent release of autoantibodies against COLVII into circulation.^7^ The synthesis of anti-COLVII autoantibodies can be inhibited by blocking heat shock protein 90 (HSP 90).^20^

In mice susceptible to the disease, polarization towards the Th1 immune response occurs, with increased production of IFN-γ and IL-4 in the peripheral lymph nodes at the immunization site.^20^

The tissue lesion is triggered by the autoantibody deposition at the dermo-epidermal junction through binding to the COLVII epitope, with complement system activation and release of pro-inflammatory cytokines^7^ and neutrophil chemotaxis.^21^

Neutrophils bind to the Fc domain of anti-COLVII^22^ autoantibodies and initiate a signaling cascade that involves the activation of the retinoid-related orphan receptor (ROR) α, heat shock protein HSP 90, phosphodiesterase 4, phosphatidylinositol- 4,5-bisphosphate 3-kinase (PI3K), among other molecules.^7^ Thus, neutrophils are activated, secreting reactive oxygen species and proteases.^19^ These substances lead to a reduction in anchoring fibrils, with the subsequent formation of bullae on the skin and mucous membranes.^22^

Different cytokines have been linked to EBA pathogenesis, such as CXCL1, CXCL2, GM-CSF, and IL-1α/β, which show increased expression and are associated with bulla formation in experimental EBA.^19^ On the other hand, the role of IL-6 has not yet been fully clarified. Increased tissue and serum IL-6 levels are correlated with EBA activity. However, mice that do not express IL-6 show a protective effect after immunization and do not develop the disease.^23^

Data regarding the pathogenesis of non-inflammatory EBA are scarce. Different mechanisms have been proposed, such as that autoantibody bound to COLVII affects the latter, disturbing interactions with extracellular matrix proteins in the BMZ, such as type IV collagen and fibronectin.^24^ Another possibility is that autoantibodies directly interfere with the formation of the antiparallel dimer of COLVII, destabilizing the anchoring fibrils.^25^

## Clinical aspects and classification

EBA is characterized by the presence of tense bullae, erosions and skin fragility. Because they originate in the lower part of the basement membrane zone, the bullae are usually quite tense and usually last for several days. They may have clear or hemorrhagic contents.^7^ The presence of milia after the re-epithelialization of the lesions is a frequent finding in all forms of EBA, and their finding is relevant for considering of this diagnostic possibility, which will be confirmed or excluded by the clinical-histopathological-laboratory correlation.

The disease has two main clinical forms: inflammatory and mechanobullous (classical or non-inflammatory), with the inflammatory form being the most frequent one.^26^, ^27^

### Mechanobullous/classical/non-inflammatory EBA

**Figure 1:**
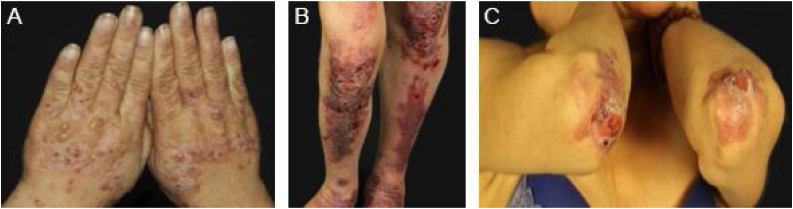
Mechanobullous epidermolysis bullosa acquisita (EBA). (A), Vesicles and bullae on the dorsum of the hands. (B), Erosions and hypertrophic scars on the knees and pretibial region. (C), Erosions and atrophic scars with milia on the elbows.

In the mechanobullous form of EBA, skin fragility and vesiculobullous lesions occur in areas that are more subject to pressure and trauma, especially the extensor surfaces of the acral regions (hands, feet, elbows, knees, pretibial region). The lesions usually appear over normal skin, without edema or erythema. They appear soon, or at most a few hours after trauma to the skin, which can be minimal. Mucous lesions are frequent. Another clinical characteristic of this form is that, during disease evolution, milia, atrophic scars, hyper- or hypopigmentation, nail dystrophy and loss, cicatricial alopecia, digital contractures, and esophageal stenosis may develop (Fig. 1).^27^

### Inflammatory EBA

**Figure 2:**
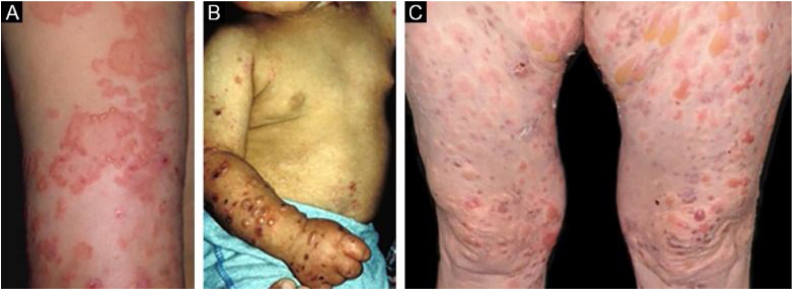
Inflammatory epidermolysis bullosa acquisita (EBA). (A), Circular and arcuate erythematous plaques with vesicles and bullae on the arm. (B), Childhood EBA. (C), Erythematoedematous papules and bullae on the thighs.

In the inflammatory form, lesions occur throughout the skin, not only in areas most often subject to trauma, and skin fragility is not so important. It may, therefore, resemble other subepidermal autoimmune bullous dermatoses, such as bullous pemphigoid (BP), mucous membrane pemphigoid (MMP), linear IgA bullous dermatosis, and Brunsting-Perry pemphigoid.^26^, ^27^, ^28^ The appearance of scars and milia during disease evolution is less frequent than in mechanobullous EBA (Fig. 2).^27^, ^28^

#### Bullous pemphigoid (BP)-like EBA

This is the most frequent subtype of the inflammatory form, and courses with tense vesiculobullous lesions on urticarial, erythematous-pruritic plaques on any part of the skin, including the face, and may affect the oral mucosa. Similar to BP itself, there may be areas where only urticarial plaques are observed, without the presence of bullae. The picture can be indistinguishable from that of BP, regarding both clinical and laboratory aspects, as both show subepidermal bullae on histopathological examination, whereas direct immunofluorescence shows linear deposition of C3 and IgG in the basement membrane zone.^28^ Patients sometimes also have lesions suggestive of mechanobullous EBA.^29^ The lesions may result in atrophic scars and milia after resolution, although they are less frequent than in the classic form.

#### Mucous membrane pemphigoid-like EBA

**Figure 3:**
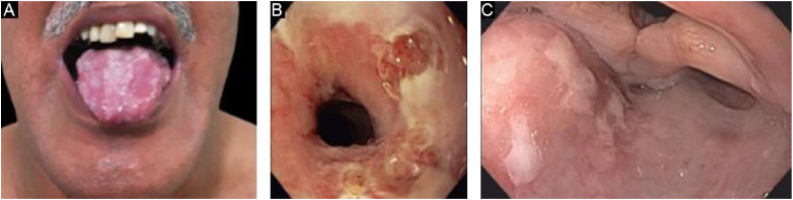
Mucosal involvement in epidermolysis bullosa acquisita. (A), Erosions on the dorsum of the tongue. (B), Bullae and erosions on the esophageal mucosa. (C), Erosions on the posterior wall of the hypopharynx.

It affects mainly the mucous membranes, such as the mouth, pharynx, esophagus, conjunctiva, anus, genital region, and respiratory tract (trachea and bronchi).^13^, ^27^, ^28^, ^30^ Only one of these sites may be affected for a prolonged period, making the diagnosis difficult. Different from what occurs in MMP, in MMP-like EBA, the mucous bullae may be long-lasting and remain intact at the time of physical examination. Cicatricial lesions (atrophic scars, synechiae, and stenoses) are identical to those that occur in MMP. These cicatricial lesions sometimes have milder consequences in the oral, genital, and anal mucosa, but can result in significant functional impairment in the esophagus, larynx, trachea, bronchi, and conjunctiva, which can lead to symblepharon, trichiasis, and even loss of vision.^31^, ^32^ Esophageal stenosis usually occurs in its upper portion and leads to dysphagia, weight loss, malnutrition, and even lung infection from food aspiration.^30^, ^32^, ^33^, ^34^, ^35^ Major lesions in the respiratory tract can lead to nasal septum perforation, pharyngeal and laryngeal stenosis, and, more rarely, tracheal and bronchial stenosis, which can lead to asphyxia.^36^ A multidisciplinary approach is mandatory in these cases (Fig. 3).

#### Linear IgA dermatosis-like EBA (IgA EBA)

It is characterized by the linear deposit of IgA in the BMZ, on its dermal side. It clinically resembles linear IgA bullous dermatosis, with the presence of annular lesions (rosette), few scars, and milia. However, it rarely progresses with the formation of mucosal scars, including a significant ocular damage.^27^, ^37^ It may show a therapeutic response to dapsone, similar to what is observed in linear IgA bullous dermatosis.^26^, ^37^

#### Brunsting-Perry MMP-simile EBA

Lesions are located only on the skin and restricted to the head, neck, and shoulder regions. The disease may course with persistent erosions and atrophic scars.^27^, ^28^, ^38^, ^39^

There have been few published EBA case series that analyzed the relative frequency of each of the clinical forms of EBA.^26^, ^31^ It is important to note that the diagnostic criteria used may vary between publications. The two most frequent forms of the disease are mechanobullous EBA and BP- like EBA. The clinical form may vary in the same patient over time; for instance, from the BP-like to the mechanobullous form.^7^, ^12^ EBA can also occur in children, and in this population, the inflammatory form also seems to be the most frequent one. The oral mucosa is more affected and the response to treatment seems to be better than in the adult population.

In general, EBA has a very important impact on patients quality of life. This can be measured by the specific scores for autoimmune bullous diseases, the ABQOL (Autoimmune Bullous Disease Quality of Life) and TABQOL (Treatment-Based Autoimmune Bullous Disease Quality of Life).^40^, ^41^

### EBA-associated Diseases

Several systemic diseases have been found in association with EBA, such as amyloidosis, thyroiditis, multiple endocrinopathy syndrome, rheumatoid arthritis, pulmonary fibrosis, chronic lymphoid leukemia, thymoma, and diabetes mellitus. However, most of them were described in isolated reports.^7^, ^12^ The only indisputable association is of EBA with inflammatory bowel disease (IBD), particularly Crohn's disease. This association is observed in approximately 25% of patients with EBA in the USA, being rarer in other countries and that is probably due to the presence of type VII collagen in the BMZ of the large intestine wall. IBD precedes the onset of EBA in most patients.^26^, ^42^, ^43^

## Diagnosis

As EBA has polymorphic mucocutaneous characteristics of varying severity, it can be mistaken with any other subepidermal autoimmune bullous dermatosis, such as bullous pemphigoid, mucous membrane pemphigoid, linear IgA bullous dermatosis, and bullous systemic lupus erythematosus (BSLE). The diagnosis is based not only on the clinicopathological correlation but also requires demonstration of the presence of *in situ* and/or circulating IgG autoantibodies against COLVII.^16^

## Histopathology

**Figure 4:**
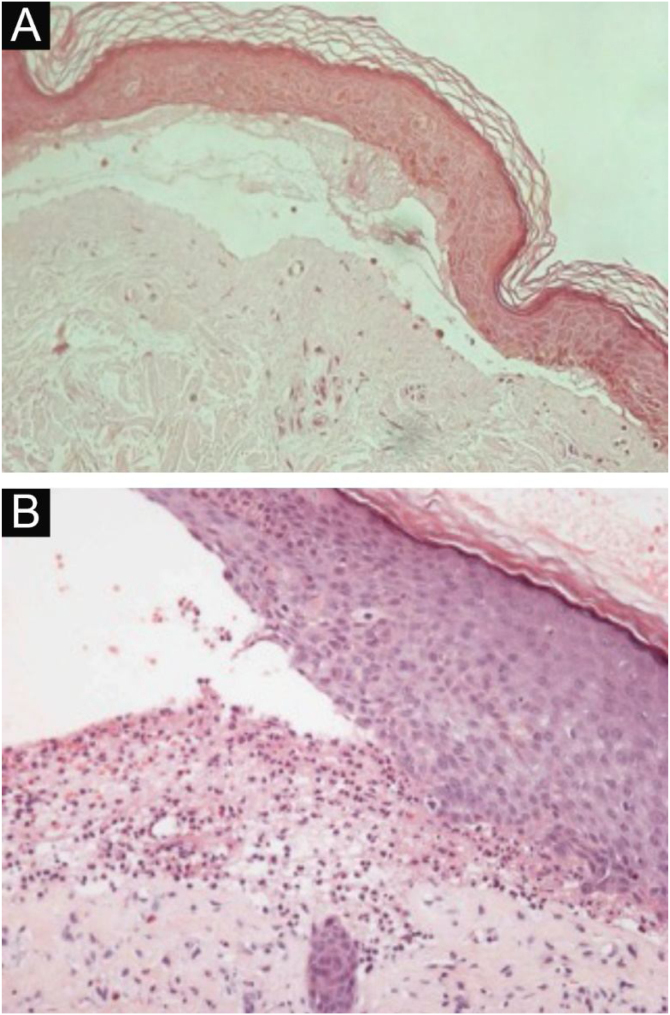
Anatomopathological examination of epidermolysis bullosa acquisita with hematoxylin-eosin staining. (A), Mechanobullous form, with subepidermal and scarce inflammatory infiltrate (×200). (B), Inflammatory form, with dermoepidermal cleavage and a rich neutrophilic perivascular inflammatory infiltrate (×400).

The histopathological findings in EBA vary according to the lesion type and duration.^16^ In classic or mechanobullous EBA, subepidermal cleavage with papillary edema and scarce inflammatory infiltrate can be seen (Fig. 4).^7^, ^44^ On the other hand, non-classical or inflammatory EBA shows intense inflammatory infiltrate with neutrophils, eosinophils, and lymphocytes in the papillary/superficial dermis (Fig. 4).^44^ Late lesions often present with keratin cysts (milia) and dermal fibrosis.^7^

## Autoantibodies *in situ*

### Direct immunofluorescence

**Figure 5:**
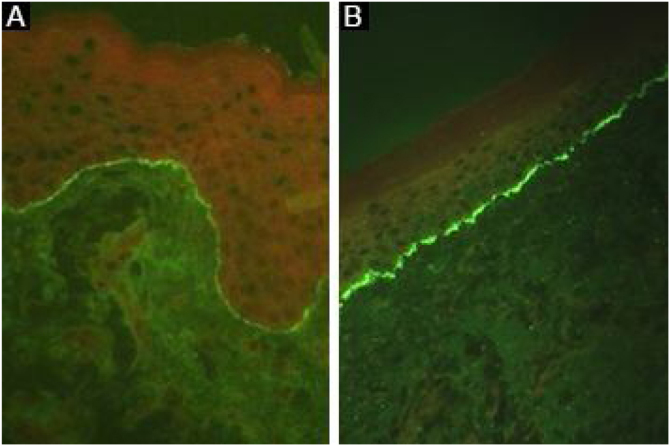
Direct immunofluorescence in epidermolysis bullosa acquisita. Intense and continuous linear fluorescence at the basement membrane zone with (A), anti-IgG and (B), anti-C3 (×400).

Linear deposits of IgG and C3 at the BMZ are present in the perilesional skin^8^ in 93% of the patients.^16^ These findings are not unique to EBA and can also be found in other subepidermal autoimmune bullous dermatoses (ABD).^45^ Fluorescence with anti-C3 is observed in 89% of the cases, followed by anti-IgG in 79% of the cases;^45^ IgA (47%) and IgM (21%) are less frequently observed (Fig. 5).^44^, ^45^ Exclusive IgA deposits can be found in 2.4% of cases corresponding to IgA-EBA.^13^

It has already been described that the analysis of the immune complex deposit pattern can increase the sensitivity of DIF:^16^ a u-serrated fluorescence pattern suggests the presence of autoantibodies bound to COLVII of the anchoring fibrils, while the n-serrated fluorescence pattern indicates the identification of antigens located above the lamina densa, such as BP180, p200, laminin 332, and laminin γ1.^8^ However, pattern analysis requires training for adequate assessment and is not widely available.

The salt-split skin technique can be performed on the fragment obtained from lesional skin, revealing fluorescence on the dermal side of the cleavage. However, this analysis is most commonly performed by indirect immunofluorescence, as incubation of the lesional skin fragment with 1 M NaCl can damage the specimen and impair the detection of immune complex deposits, reducing test sensitivity.^46^

### Immuno-electron microscopy

**Figure 6:**
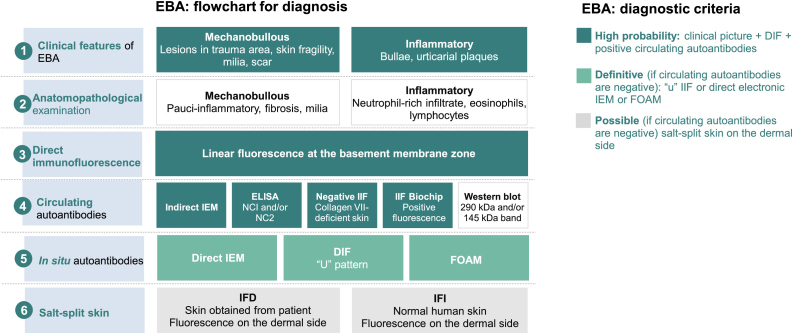
Diagnostic criteria for epidermolysis bullosa acquisita (EBA).^8^ DIF, Direct Immunofluorescence; IIF, Indirect Immunofluorescence; NC, non-collagenous domain; IEM, immuno-electron microscopy; FOAM, Fluorescence Overlay Antigen Mapping. The flowchart for the diagnosis of EBA is based on the correlation between the dermatological examination and the results of complementary studies since there is no single clinical or laboratory finding that can allow diagnostic confirmation. Currently, it is suggested to consider EBA as a diagnostic hypothesis in cases with skin fragility, scar formation, and milia (mechanobullous form) or urticarial plaques with bullae (inflammatory form). The clinical picture correlates with the anatomopathological findings: the mechanobullous form shows a pauci-inflammatory subepidermal cleavage, whereas in the inflammatory form a neutrophil-rich- dermo-epidermal bullous dermatosis can be observed. The autoimmune nature of the disease can be confirmed by direct immunofluorescence, which allows the detection of immune complex deposits, mainly IgG, followed by C3, IgA, and IgM. Moreover, the screening for circulating autoantibodies can be performed using different diagnostic methods such as indirect immuno-electron microscopy, ELISA, indirect immunofluorescence (normal stratified squamous epithelium substrate or biochip), and Western blot. When the previously mentioned tests are negative or inconclusive, *in situ* autoantibody screening can be performed by direct immuno-electron microscopy, direct immunofluorescence serrated pattern, or fluorescent overlay antigen mapping (FOAM). However, as these techniques are more available for research purposes, direct or indirect immunofluorescence with the salt-split skin technique is commonly used in clinical practice to demonstrate immune complex deposits on the dermal side of the cleavage, where collagen VII is located.

Demonstration of IgG, C3, and IgA^47^ deposits in the anchoring fibrils below the lamina densa^8^, ^16^ using direct immuno-electron microscopy remains the gold-standard technique for diagnosing EBA.^44^ As this method is unavailable in most institutions, additional studies are recommended to confirm the diagnosis of EBA (Fig. 6).

### Fluorescent Overlay Antigen Mapping (FOAM)

This technique compares the location of a known BMZ antigen to the immune complexes deposited in perilesional skin obtained from patients with EBA,^7^ using immunostaining with different fluorescent colors.^8^

### Immunohistochemistry

Formalin-fixed and paraffin-embedded fragments obtained from the lesional skin of patients with EBA are stained with anti-collagen IV, which is located in the lamina densa. As the level of cleavage in EBA occurs below the lamina densa, collagen IV will be positive at the roof of the bulla.^8^

## Circulating autoantibodies

Circulating autoantibodies in EBA can be demonstrated in about 50% of patients and correlate with disease severity.^48^ The sensitivity varies according to the methodology used, as described below.

### Indirect immunofluorescence

The presence of circulating anti-COLVII IgG can be demonstrated using serum samples obtained from patients with EBA. After incubation with a sample of normal skin, monkey esophagus, or rat bladder,^16^, ^44^ the deposits of immune complexes at the BMZ are seen as linear fluorescence in up to 37% of patients.^44^ Anti-IgA positivity usually occurs at low titers (1:2 to 1:320)^37^ and is seen in 2.3% of cases.^13^

**Figure 7:**
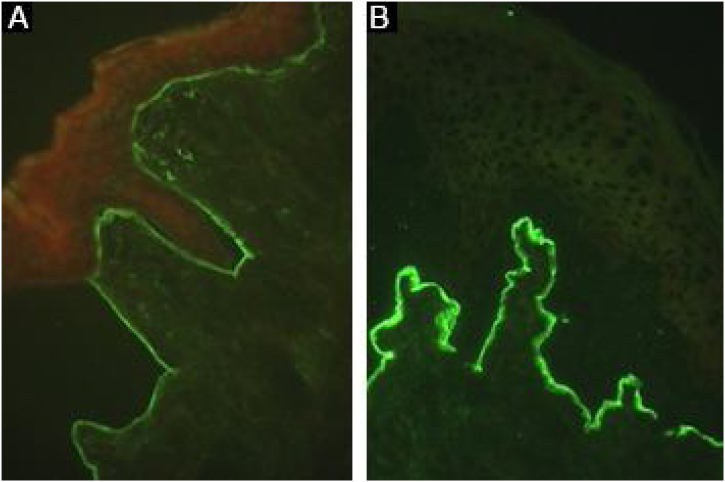
Indirect immunofluorescence in epidermolysis bullosa acquisita. (A), Strong linear fluorescence on the basement membrane zone with anti-IgG (×400). (B), Fluorescence on the dermal side of the cleavage with the salt-split skin technique using anti-IgG (×400).

Sensitivity can be increased up to 74.7% using the salt-split technique,^8^ in which normal human skin is cleaved at the level of the lamina lucida with 1 M NaCl, enhancing the exposure of hemidesmosome antigens. As COLVII remains on the dermal side of the cleavage, incubation with serum from EBA patients will produce a linear fluorescence on the floor of the bulla (Fig. 7).^48^

Skin fragments showing a lower expression of COLVII, obtained from patients with dystrophic epidermolysis bullosa can also be used as a substrate. In patients with EBA, the incubation of preselected positive sera on normal human skin will result in negative or less intense fluorescence after incubation with a skin sample from a patient with dystrophic epidermolysis bullosa.^8^

The IgG subclass analysis may provide additional information for the differentiation between EBA and BSLE, since COLVII is the target antigen in both diseases; IgG1 and IgG4 are seen in EBA, while IgG2 and IgG3 are usually detected in BSLE.^49^

### ELISA

Commercially available ELISA systems containing non-collagenous (NC) 1 and/or NC2 epitopes can be used to demonstrate and quantify the presence of IgG autoantibodies against COLVII.^16^ Sensitivity has been reported between 20% to 98%^44^, ^50^ and varies depending on the type of NC recombinant protein used and the selection of known positive serum samples from patients with EBA.^8^ Positivity correlates with disease activity;^50^ however, it does not correlate with the clinical presentation (mucous vs. cutaneous involvement).^7^

### Western blot

Different epitopes of COLVII can be obtained from recombinant proteins and cell or tissue extracts to produce a 290 kDa and/or 145 kDa band.^44^, ^47^ The sensitivity varies from 20% to 80%, according to the COLVII source and the type of NC domain used.^44^ False-negative results may occur due to conformational changes in epitopes during the preparation of the recombinant proteins.^51^

### Biochip

Circulating anti-COLVII IgG can be detected after incubation of the serum with human cells transfected with the NC1 domain. Immune complex formation is revealed using a fluorescein-labeled secondary anti-human antibody, with sensitivity and specificity similar to those described for ELISA studies.^8^

## Complementary exams

The dermatologist plays a key role in coordinating the multidisciplinary follow-up of patients with EBA. Screening for mucosal involvement in EBA is crucial to assess the presence of active lesions and/or sequelae and to determine the best therapeutic approach according to disease severity (Table 1).^16^, ^47^ It is also recommended to evaluate the association with Crohn's disease, as well as comorbidities.^47^

**Table 1:** Recommended screening for patients with epidermolysis bullosa acquisita: assessment of mucosal involvement.^16^, ^47^

**Mucosal involvement**	Nasal and oropharyngeal	Nasofibroscopy
	Esophageal	Upper gastrointestinal endoscopy
	Gynecological	Vulvoscopy
		Colposcopy
	Ocular	Eye examination

## Differential diagnosis

Mechanobullous EBA may be clinically indistinguishable from porphyria cutanea tarda (PCT), as both diseases show skin fragility with bulla formation and scarring in areas susceptible to trauma. Hypertrichosis, liver dysfunction, and increased ferritin and porphyrin levels can be seen in PCT and contribute to its diagnosis.^52^ The anatomopathological examination is characterized by the presence of festooning of the dermal papillae and thickening of the vascular wall, especially with periodic acid-Schiff (PAS) staining. Homogeneous deposits of IgG, IgM, IgA at the BMZ and vessel wall are the most common DIF findings in PCT and help in its differentiation from EBA.^53^

When patients have an early-onset of skin fragility (congenital) or have a family history of epidermolysis bullosa (EB), the diagnosis of hereditary dystrophic EB must be ruled out.^47^ In congenital EB, DIF will be negative, whereas immune mapping will reveal a decrease or absence of COLVII.^54^

Inflammatory EBA may present with pruritic urticarial plaques and bullae, as in bullous pemphigoid.^7^ The anatomopathological examination in bullous pemphigoid can help in the diagnostic differentiation, due to the presence of eosinophilic spongiosis or a subepidermal bullous dermatitis with eosinophils. The most frequent DIF findings are the presence of linear deposits of C3 and IgG in the BMZ, whereas IFI, with the salt-split skin technique, allows the differentiation from EBA, since in bullous pemphigoid, fluorescence occurs on the epidermal or epidermal and dermal sides of the cleavage.^55^

Patients with EBA may show predominantly mucosal involvement, with healing with scarring formation in the eyes, mouth, nose, pharynx, larynx, esophagus, urethra and/or anus. The subsequent sequelae resemble those seen in MMP or linear IgA bullous dermatosis.^47^ Histopathology of MMP is commonly characterized by a pauci-inflammatory dermoepidermal cleavage, whereas in linear IgA bullous dermatosis neutrophilic subepidermal bullous dermatitis is observed. On DIF, MMP may be indistinguishable from other forms of pemphigoid and EBA. For this reason, IIF with the salt-split skin technique contributes to diagnostic differentiation: while fluorescence is observed on the epidermal side of the cleavage in most cases of MMP, in EBA the fluorescence is observed on the dermal side of the cleavage. However, in cases of anti-p200 MMP and anti-laminin 332, fluorescence is also observed on the dermal side of the cleavage.^56^ The cases of linear IgA bullous dermatosis and EBA with exclusive deposition of linear IgA on the BMZ on DIF can be differentiated by IIF with the salt-split skin technique. In linear IgA bullous dermatosis, the fluorescence occurs on the epidermal side of the cleavage, whereas in EBA this fluorescence is seen on the dermal side.^55^

Bullous systemic lupus erythematosus (BSLE) is another potential differential diagnosis when tense bullae arise primarily on sun-exposed areas of female patients with photosensitivity and usually progress without scar formation or milia.^57^ The histopathological and DIF and IIF findings are similar in BSLE and EBA, since both diseases show the formation of anti-COLVII antibodies. Laboratory findings that confirm the diagnosis of systemic lupus, such as positive antinuclear autoantibodies and extracutaneous manifestations, such as renal, hematological, articular, and neurological involvement, constitute criteria that help to differentiate between BSLE and EBA.^57^

## Treatment

EBA treatment aims to control disease activity and prevent recurrence and permanent sequelae. Disease control is defined as the cessation of the appearance of new lesions and the healing of pre-existing ones.^58^ It is crucial to assess the degree of mucocutaneous involvement, the presence of sequelae, patient age, and comorbidities to determine the therapeutic choice.

The prevention of new lesions includes patient education to (1) Protect the skin from additional trauma by using soft fabric clothing and non-adherent dressings, (2) Perform adequate cleaning of lesions with soap and water, (3) Avoid eating foods that may cause additional damage to the oral mucosa during chewing and swallowing, such as hot drinks and acidic, rough/crunchy products, (4) Adhere to the proposed treatment and follow-up visits to make early adjustments of therapy, if necessary.

Frequent reassessment of mucosal involvement by a specialized multidisciplinary team is also important for an early diagnosis of new lesions, prevention of scar formation, and introduction of appropriate treatment.^16^ Sequelae may require surgical treatment including oculoplastic surgery, removal of nasal, urethral, ​​and gynecological synechiae, esophageal dilation, or gastrostomy, if there is severe stenosis and tracheostomy in case of laryngeal involvement with lumen reduction and airway obstruction.^59^

**Figure 8:**
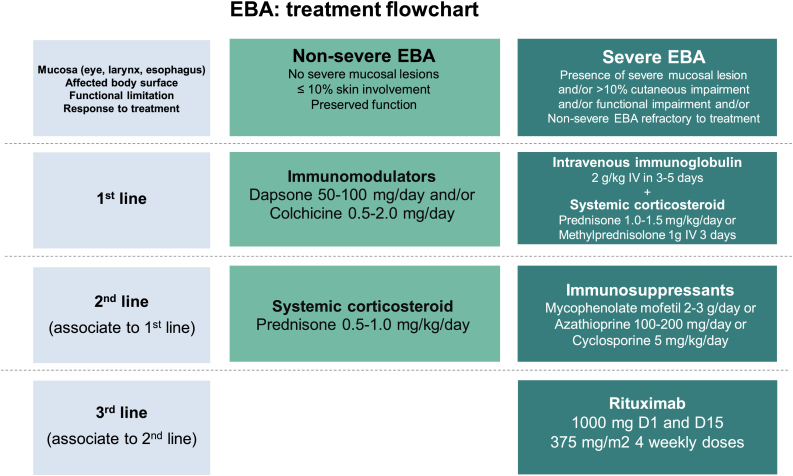
Treatment of epidermolysis bullosa acquisita (EBA).

Due to the rarity of this autoimmune bullous dermatosis and diverse clinical manifestations, randomized controlled studies regarding the treatment of EBA are scarce. Current recommendations are based on case and series reports, as well as expert opinions obtained from national and international consensus and guidelines.^47^, ^59^, ^60^, ^61^ Most of them suggest that the choice of therapy should be programmed according to disease severity, which can be objectively assessed by the Bullous Pemphigoid Disease Area Index (BPDAI) or subjectively by the degree of mucosal involvement and risk of long-term sequelae and functional limitations.^47^, ^59^ Thus, exclusively mild cutaneous manifestations could be treated with systemic corticosteroids and immunomodulators, while ocular, laryngeal, esophageal, and urethral involvement requires the use of systemic corticosteroid therapy associated with immunosuppressants and/or rituximab and IVIg for disease control (Fig. 8).^60^

### Systemic corticosteroids

Oral, intravenous, or corticosteroid pulse therapy are considered the first choice of treatment for EBA, although current evidence suggests that it promotes better control, especially of the inflammatory form of the disease.^62^ The precise mechanism of action of corticosteroids in EBA has not yet been fully understood. It is known that corticosteroid therapy induces neutrophilia and reduces cytokine release and levels of lymphocytes, eosinophils, and monocytes.^63^ Experimental models demonstrate that methylprednisolone acts on neutrophils, inhibiting the phosphorylation of kinases such as p38 MAPK, ERK 1/2 and Akt. The inactivation of these pathways results in reduced synthesis of reactive oxygen species and neutrophil degranulation, with a subsequent decrease in subepidermal cleavage.^64^

The recommended initial dose ranges from 0.5 mg/kg/day in mild cases to 1.5 mg/kg/day in severe ones. For patients with dysphagia, oral corticosteroid solutions may be more effective and tolerable than tablets. As prolonged systemic corticosteroid therapy is related to several complications including ocular (cataract, glaucoma), metabolic (obesity, diabetes, hypertension, dyslipidemia, osteoporosis, Cushing's syndrome), and osteoarticular ones (femoral head avascular necrosis), immunosuppressants, immunomodulators and rituximab are used due to their corticosteroid-sparing effect.^63^

### Dapsone (DDS)

DDS at a dose of 25‒150 mg/day reduces neutrophil chemotaxis and has a corticosteroid-sparing effect.^8^ DDS can even be used in monotherapy with adequate control of IgA-mediated EBA activity^7^ and in pediatric patients.^8^, ^62^ Disease response is usually observed within 2 weeks after the onset of treatment.^62^ Due to potential adverse effects such as hemolytic anemia, methemoglobinemia, agranulocytosis, and DRESS (drug reaction with eosinophilia and systemic symptoms), frequent laboratory monitoring is required. Assessment of glucose-6-phosphate dehydrogenase levels is also recommended, as low levels correlate with hemolysis.

### Colchicine

Mild EBA can be controlled using colchicine 1‒2 mg/day alone or combined with systemic corticosteroid therapy, with few adverse effects such as dose-dependent diarrhea.^7^ Colchicine is beneficial in patients in which immunosuppressants should be avoided, as it inhibits neutrophil chemotaxis and increases prostaglandin E2.^65^

### Cyclosporine (CyA)

CyA at a dose of 4 to 9 mg/kg/day was rarely used as an adjuvant treatment in EBA, although the improvement was reported in 11/11 patients.^16^ Nephrotoxicity, as well as other potential adverse effects such as hypertension, hypertrichosis, dyslipidemia, and headache^7^ may limit the use of CyA.

### Mycophenolate mofetil (MMF)

MMF at a dose of 2 to 3 g/day has been successfully used in combination with systemic corticosteroid therapy to promote disease remission and even as monotherapy after complete corticosteroid tapering.^66^ Most data regarding the efficacy and safety of MMF come from studies including patients with pemphigus vulgaris or bullous pemphigoid,^7^ and its specific mechanism of action in EBA remains unknown. As MMF acts on lymphocyte purine synthesis,^67^ the resulting depletion of B cells possibly reduces the synthesis of autoantibodies against COLVII.^66^ Nausea, diarrhea, hepatitis, and lymphopenia have been reported as common adverse effects.^67^ Additional studies are required to better clarify the role of MMF in the treatment of EBA.

### Azathioprine (AZA)

AZA at a dose of 2 to 3 mg/kg/day may be combined with systemic corticosteroid therapy in moderate to severe EBA.^7^ The immunosuppressive effect involves the inhibition of nucleic acid and protein synthesis, with depletion of mononuclear cells and lymphocytes.^67^ The activity of thiopurine methyltransferase interferes with the metabolism of AZA, and the measurement of its levels can be useful for evaluating the treatment dosage and for preventing dose-dependent adverse effects, such as hepatitis and leukopenia.^7^, ^67^ Additional idiosyncratic adverse effects include nausea, pancreatitis, and diarrhea.^7^ Pre-treatment evaluation and regular laboratory tests are recommended (Table 2).

**Table 2:** Main drugs used in the treatment of epidermolysis bullosa acquisita.^7^, ^8^, ^9^, ^25^, ^26^, ^27^, ^28^, ^29^, ^30^, ^31^, ^32^, ^33^, ^63^, ^65^, ^67^, ^71^, ^72^, ^73^, ^74^, ^75^, ^76^

Drug (dose)	Main action mechanisms	Main adverse effects	Laboratory evaluation
Systemic corticosteroid (prednisone 0.5‒1.0 mg/kg/day)^a^ or (prednisone 1.0‒1.5 mg/kg/day)^b^ or (methylprednisolone 1 g/day IV for 3 days)^b^	Inhibition of cytokines, cytopenias (eosinophils, lymphocytes, monocytes), neutrophilia	Ocular (cataract, glaucoma), metabolic (obesity, diabetes, hypertension, dyslipidemia, osteoporosis, Cushing's syndrome), osteoarticular (femoral head avascular necrosis)	Complete blood count, liver enzymes, renal function, fasting glucose, glycated hemoglobin, total cholesterol and fractions, triglycerides, bone densitometry
Dapsone^a^ (50‒100 mg/day)	Anti-neutrophilic action, with reduced chemotaxis of neutrophils	Hemolytic anemia, methemoglobinemia, agranulocytosis, DRESS (drug reaction with eosinophilia and systemic symptoms)	Glucose-6-phosphate dehydrogenase, complete blood count, liver enzymes, lactate dehydrogenase, reticulocytes, total bilirubin and fractions
Colchicine^a^ (0.5‒2.0 mg/day)	Anti-neutrophilic action, with inhibition of neutrophil chemotaxis and increase in prostaglandin E2	Neutropenia, diarrhea, abdominal discomfort	Complete blood count, liver enzymes, kidney function
Cyclosporine^b^ (5 mg/kg/day)	Calcineurin phosphatase inhibition, causing depletion of T cells and macrophages, and activation of natural killer cells, T cells, and antigen-presenting cells	Nephrotoxicity, hypertension, hypertrichosis, dyslipidemia, headache	Complete blood count, liver enzymes, kidney function, total cholesterol and fractions, triglycerides
Mycophenolate mofetil^b^ (2‒3 g/day)	Inhibition of purine synthesis, causing lymphocyte depletion	Nausea, diarrhea, hepatitis, lymphopenia	Complete blood count, liver enzymes, kidney function, serology for hepatitis B, C, HIV
Intravenous immunoglobulin (2 g/kg IV in 3‒5 days)	Depletion of autoantibodies by reducing the half-life of immunoglobulins	Headache, chest pain, fever, dyspnea, myalgia, nausea, vomiting, diarrhea, tachycardia, erythema, anaphylaxis, acute kidney injury, thromboembolism, aseptic meningitis, neutropenia, hemolytic anemia	Complete blood count, renal function, liver enzymes, serum immunoglobulin levels (to rule out IgA deficiency, due to the increased risk of anaphylaxis)
Rituximab^b^ (1000 mg D1 and D15 or 375 mg/kg/m^2^ four weekly doses)	Chimeric anti-CD20 monoclonal antibody that induces B lymphocyte depletion by inducing apoptosis, complement activation and cytotoxicity	Fever, nausea, vomiting, angioedema, bronchospasm, anaphylaxis, infection, hepatitis B reactivation, angina, arrhythmia, heart failure, coronary syndrome	Complete blood count, liver enzymes, kidney function, serology for hepatitis B, C, HIV

a Non-severe EBA: absence of mucosal, ocular, laryngeal, esophageal lesions and ≤10% of the affected body surface without functional limitation.

b Severe EBA: the presence of ocular, laryngeal, esophageal lesions and/or >10% of the affected body surface and/or the functional limitation or non-severe EBA refractory to treatment.

### Cyclophosphamide (CyP)

The literature regarding the use of oral CyP (50 to 100 mg/day) or intravenous pulse therapy (500 to 1,000 mg/m^2^) is scarce.^7^, ^61^ The combination of CyP and corticosteroid pulse therapy has been reported as an attempt to achieve disease control in patients with severe mucosal involvement. CyP inhibits DNA synthesis and induces apoptosis. However, myelosuppression, hemorrhagic cystitis, infertility, and carcinogenesis^61^ have limited the use of CyP, particularly after the advent of rituximab.^67^

### Methotrexate (MTX)

MTX at a dose of 20 to 25 mg/week is used in combination with systemic corticosteroid therapy as a corticosteroid-sparing agent, as it inhibits nucleic acid synthesis and lymphocyte activation.^68^ Few reports regarding the use of MTX in EBA have been published; therefore, it is not possible to adequately assess drug efficacy to achieve disease control.^7^ Common adverse effects include alopecia, cytopenias, abdominal discomfort, and hepatotoxicity.^68^ Laboratory evaluation is recommended before the introduction of MTX and throughout treatment (Table 2).

### Intravenous immunoglobulin (IVIg)

IVIg at a dose of 2 g/kg over 3 to 5 consecutive days is an alternative treatment for severe and recalcitrant EBA,^7^ as it reduces pathogenic autoantibodies.^69^ According to a recent meta-analysis on the treatment of EBA, only IVIg treatment was associated with the complete remission of inflammatory EBA.^13^ However, IVIg-treated EBA patients require 16 to 22 cycles to achieve disease control, which limits its use due to the high cost of the treatment.^58^ IVIg is also useful in combination with rituximab (RTX) to reduce the risk of infection after anti-CD20 infusion.^69^ The most common adverse effect of IVIg is headache.^7^

### Plasmapheresis/Immunoadsorption

The removal of circulating autoantibodies in combination with rituximab treatment has been reported for the management of recalcitrant EBA cases.^7^ However, plasmapheresis and immunoadsorption are not widely available and, therefore, have been used in a few cases.

### Rituximab (RTX)

Complete remission of EBA has been reported with the use of RTX, an anti-CD20 chimeric monoclonal antibody. The most commonly used treatment regimens comprise 1,000 mg/infusion on D1 and D15 (rheumatoid arthritis protocol) or 375 mg/m^2^/week for 4 weeks (lymphoma protocol).^7^

A literature review carried out in 2018 evaluated the treatment of EBA with RTX alone or in combination with IVIg or immunoadsorption. Of 16 patients treated with RTX in monotherapy at a dose of 500 or 1,000 mg on D1 and D15, 13 responded initially (three with partial response, three with complete response, five with disease remission, one death from pneumonia on D21, and one was lost to follow-up) and three showed no response. The combination of RTX 375 mg/m^2^/week for 4 weeks with IVIg 2 g/kg induced clinical improvement and disease control in 5/5 patients. However, due to the persistence of skin lesions in 4/5 patients, maintenance treatment with IVIg once a month was required. RTX (lymphoma protocol) and immunoadsorption were used in five patients, two of them achieved disease control. As plasma filtration with immunoglobulin removal and reinfusion is not a widely available technique, it is not possible to assess the efficacy of immunoadsorption in EBA to date.^70^

Patients with EBA commonly have recurrences during the course of the disease, sometimes with conventional treatment refractoriness. A meta-analysis identified 1,159 cases of EBA reported from 1971 to 2016 and assessed which drug induced a complete response when used in monotherapy.^13^ Most patients with EBA used multiple medications, due to the ineffectiveness of previous treatments. Despite study limitations, including its retrospective design, limited number of case reports, and heterogeneity of treatments and outcomes, the authors concluded that intravenous immunoglobulin and rituximab were associated with clinical disease remission. The subgroup analysis, however, demonstrated that the response to treatment among the EBA variants was distinct: intravenous immunoglobulin was associated with complete remission (CR) in mechanobullous EBA, whereas no drug therapy was associated with CR in inflammatory EBA.

### Future perspectives in the treatment of EBA

Discoveries about the disease pathogenesis allowed the characterization of potential therapeutic targets including immunobiological ones that inhibit the complement system activation (anti- gamma receptor fraction antibodies – FcR); proteins that decrease the expression of pro-inflammatory interleukins, such as IL-6, and stimulate the synthesis of anti-inflammatory cytokines such as IL-4 and IL-10; molecules and pathways that reduce neutrophil chemotaxis and activation (LTB4, p38 MAPK, GM-CSF).^7^, ^19^ New drugs for the treatment of EBA have been studied and developed based on experimental animal models and, therefore, their efficacy and safety in humans remain to be elucidated.

## Evolution and prognosis

**Figure 9:**
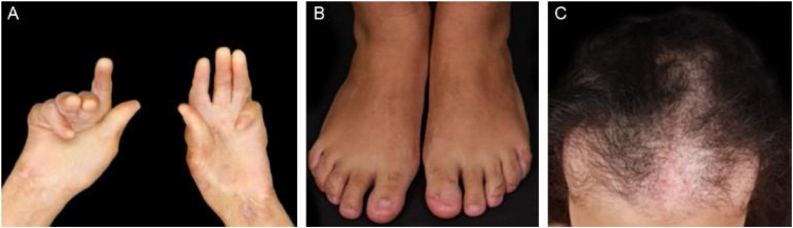
Complications on the skin and its adnexa in epidermolysis bullosa acquisita. (A), Atrophy of the palmar region with flexion contractures of the hands. (B), Anonychia. (C), Cicatricial alopecia.

**Figure 10:**
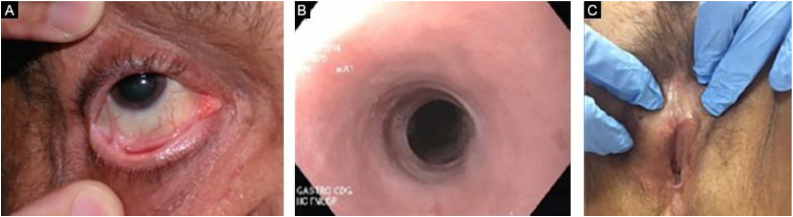
Mucosal complications in epidermolysis bullosa acquisita. (A), Conjunctival synechiae. (B), Esophageal substenosis. (C), Encapsulation of the clitoris and synechia of the labia minora.

EBA is a chronic disease with high morbidity due to its permanent scarring potential (Figure 9, Figure 10). The development of stenoses and synechiae secondary to mucosal disease activity may occur subclinically.^30^ Its delayed identification increases the potential for severe complications with a significant impact on quality of life.^77^ Overall, the prognosis and response to treatment in EBA are better in children.^78^

A multidisciplinary study with 4 EBA patients has shown that 50% of them already present airway synechiae before the onset of significant symptoms.^30^ Another study of patients with EBA^77^ showed that 5/12 patients had mucosal complications, including esophageal stenosis (2/12), and laryngeal synechiae (2/9), symblepharon and trichiasis (2/12), and hand deformity (3/12).

Although data regarding the prognostic factors of EBA are scarce in the literature, the course and prognosis of the disease are believed to be associated with the severity at the time of diagnosis and response to the proposed treatment.^7^ The correlation between disease severity and activity and serum levels of anti-COLVII autoantibodies has also been described.^50^, ^79^

In a retrospective study with 30 patients,^26^ after one year of follow-up, partial response was observed in 20.8% of the patients and 33.3% developed a complete response. The time to attain remission and the percentage of response between the mechanobullous and inflammatory forms of EBA were similar. The authors used the concept of remission for EBA based on the adaptation of the definitions described by the International Pemphigus Committee.^80^

## Financial support

None declared.

## Authors’ contributions

Denise Miyamoto: Critical review of the literature; drafting and editing of the manuscript; approval of the final version of the manuscript; critical review of the manuscript.

Juliana Olivieri Gordilho: Critical review of the literature; drafting and editing of the manuscript; approval of the final version of the manuscript; critical review of the manuscript.

Claudia Giuli Santi: Critical review of the literature; drafting and editing of the manuscript; approval of the final version of the manuscript; critical review of the manuscript.

Adriana Maria Porro: Critical review of the literature; drafting and editing of the manuscript; approval of the final version of the manuscript; critical review of the manuscript.

## Conflicts of interest

None declared.
